# Multiple Sclerosis in a Dog

**Published:** 1898-07

**Authors:** Wilfred Lellman

**Affiliations:** Professor of Materia Medica and Therapeutics, and Parasites and Parasitic Diseases, New York College of Veterinary Surgeons, New York City


### REPORTS OF CASES.[1]

### MULTIPLE SCLEROSIS IN A DOG.

By Wilfred Lellman, D.V.M.,
PROFESSOR OF MATERIA MEDICA AND THERAPEUTICS, AND PARASITES AND PARASITIC DISEASES, NEW YORK COLLEGE OF VETERINARY SURGEONS, NEW YORK CITY.

A very interesting case of paralysis in a dog was observed by me last year. When I discuss this case it is done with some reluctance, as it was impossible for me to get the animal for an autopsy in order to prove that my diagnosis was correct ; nevertheless, there is no doubt in my mind.

In September of last year I was called to examine a dog, which, according to the owner's history, had been paralyzed gradually during the past six months. When securing the history of the case as thoroughly as possible, I found out the following facts : The first symptoms which the owner of the animal noticed were an intense twitching of the muscles of the extremities when the animal got up to walk or when it became excited; during rest these symptoms would cease entirely. By and by, the owner said, she noticed the appearance of weakness in the hind legs, staggering gait, till finally the animal became entirely paralyzed in its hind legs ; the forelegs also seemed to become weaker and not to be able to perform their normal function ; the right front extremity more especially. The dog, which was trained well and used to know a good many tricks, seemed to have forgotten all but one of them entirely. The owner told me that, according to her opinion, the dog had very poor eyesight. The appetite had been fair. When asked whether any irregularities in passing the feces and urine had been noticed, the owner told me that she had never noticed any.

A thorough examination was made, the results being as follows : Male pug dog, about six years old ; in abnormal fat condition, the hair being of dull lustre, the visible mucous membranes appear to be anæmic ; pulse is small, irregular, beating about 100 times a minute. At the two brachial arteries the pulse appears not synchronous. Temperature per rectum is 102° F. The heart-shock is weak—can hardly be felt on the left side of the thorax. Percussion of the heart-region shows an increased zone of dulness. While auscultating the heart I find the first sound normal, while the second one can hardly be distinguished ; it appears to be combined with a by-sound almost synchronous with the diastole of

[1] Read before the Veterinary Medical Association of New York County, June, 1898.

the heart. This abnormal by-sound is of a somewhat buzzing character. According to my examination of the heart I diagnosed insufficiency of the semilunar valves of the aorta, and also a possible aneurism (dilatation). There is, also, most probably a dilatation of the left ventricle. Percussion of the thoracic walls shows nothing abnormal apart from the increased dulness of the heart-region. Auscultation of the lungs reveals dry bronchial râles. Examination of the digestive apparatus reveals nothing very important, except a catarrh of the intestinal tract. Disturbances in the function of the rectum are not present. Examination of the uro-genital organs proves slight albuminuria, and quite an enlargement of the prostatic gland. The pysche of the animal is not free; it appears distinctly depressed, somewhat apathetic. A thorough examination of the eyes shows considerable dilatation of the pupils, partial atrophy of the optic nerves, some small calcifications within the lens; these, however, were not sufficient to cause any substantial disturbance of the eyesight. The hind extremities are almost perfectly paralyzed. When trying to move, the dog drags the extremities; the animal is unable to make any coördinate movements with the same. The fore extremities show commencing paretic symptoms, the right one more so. However, there is not substantial atrophy of any muscles of the extremities and the trunk. Almost puzzling is the considerable atrophy of both temporal muscles. Every time the animal tries to move a considerable tremor of the muscles of the extremities sets in; during rest, however, it disappears altogether. While testing the sensibility of the nerves I can hardly detect anything abnormal. On the other hand, the reflex of the tendons and periosteum appears distinctly increased. The hind extremities show considerably increased patellar reflex, and also intense clonus of the foot (reflex of the Achillean tendon). The muscles of the posterior extremities are in a kind of tonic state. The gait of the hind extremities is paretic-spastic. The reflex of the cutis appears to be normal. Trophic lesions are not present. Disturbances in the function of the rectum and bladder are also absent. By request, some milk was given to the dog. I now notice that there were beginning disturbances in swallowing. The lower lip appears somewhat flabby and is hanging down. The voice of the dog is also changed. This was told me by the owner before. Those last-mentioned symptoms speak clearly for pathological alterations of the medulla, while atrophy of the optic nerves is due to cerebral alterations. The paraplegia of the posterior extremities I consider due to chronic alterations within the lateral and anterior tracts of the spinal cord.

It is easily understood that the clinical symptoms of multiple sclerosis of the brain and spinal cord can be very variable, according to the localization of those sclerotic alterations. In this case, according to the clinical symptoms, I localized the sclerotic alterations principally within the occipital lobes of the hemispheres, within the medulla oblongata, pons varolii, and within the spinal cord from the thoracic to the lumbar region. I base my diagnosis upon the following facts 1. Upon the slow progressive paralysis; any neo-formations would have been of a quicker course, and would have revealed other clinical symptoms besides. 2. Upon the condition of the whole system of the dog; upon the clinical examination of the heart, which proved to be alteration of the ascendent aorta, principally insufficiency, and most probably aneurism; obesity of the heart. Those reasons are strengthened by the *modus vivendi* of the animal—that is, absolutely wrong diet and lack of exercise. It is easily understood that those disturbances within the circulation are apt to cause chronic alterations of the central nervous system, due to insufficient nutrition.

The sclerosis I consider due to alterations of the very fine bloodvessels of the central nervous system, perhaps due to arteriosclerosis or fatty degeneration of the muscular coat. Those arteriosclerotic alterations are very often followed by thromboses and embolism. As the prognosis was very bad, I advised the owner to destroy the dog, which was done. It was, however, impossible for me to obtain the dog for an autopsy, as the owner did not want to have him cut up.

